# A Time to Sleep Well and Be Contented: Time Perspective, Sleep Quality, and Life Satisfaction

**DOI:** 10.3389/fpsyg.2021.627836

**Published:** 2021-04-16

**Authors:** Michael Rönnlund, Elisabeth Åström, Wendela Westlin, Lisa Flodén, Alexander Unger, Julie Papastamatelou, Maria Grazia Carelli

**Affiliations:** ^1^Department of Psychology, Umeå University, Umeå, Sweden; ^2^Ludwigshafen University of Business and Society, Ludwigshafen, Germany

**Keywords:** sleep quality, time perspective, balanced time perspective, chronotype, life satisfaction

## Abstract

A major aim of the present study was to examine the relationship between time perspective, i.e., habitual ways of relating to the past, present, and future, and sleep quality. A second aim was to test a model by which the expected negative relationship between deviation from a balanced time perspective (DBTP), a measure taking temporal biases across all three time frames into account, and life satisfaction was mediated by poor sleep quality. To these ends, a sample of young adults (*N* = 386) completed a version of the Zimbardo Time Perspective Inventory (S-ZTPI), Pittsburg Sleep Quality Index (PSQI), and the Satisfaction with Life Scale (SWLS). A measure of chronotype was in addition included for control purposes. Bivariate analyses revealed that the S-ZTPI subscales Past Negative, Future Negative and Present Fatalistic were associated with poorer sleep quality (higher PSQI scores), with significant associations in the opposite direction for Past Positive and Future Positive. However, DBTP was the strongest predictor of (poorer) sleep quality, suggesting that time perspective biases have an additive effect on sleep quality. Regression analyses with PSQI as the dependent variable and all six ZTPI subscales as the predictors indicated that time perspective accounted for about 20% of the variance in sleep quality (17% beyond chronotype), with Past Negative, Past Positive, and Future Negative as the unique predictors. The results additionally confirmed a strong relationship between DBTP and life satisfaction. Finally, data were consistent with the hypothesis that the association of DBTP and life satisfaction is mediated, in part, by sleep quality. Taken together, the results confirmed a substantial link between time perspective sleep-related problems, factors that may have a negative impact on life satisfaction.

## Introduction

Sleep is essential to homeostatic processes required for adequate human functioning, including cognitive (Deak and Stickgold, 2010) and socio-emotional functioning (Beattie et al., 2015) and sufficient sleep is critical for maintaining physical health (Medic et al., 2017). An important aspect of sleep is sleep quality, i.e., the degree to which one perceives sleep to be free from disturbances. Poor sleep quality was, for example, linked to increased risk for cardio-vascular disease (Lao et al., 2018), cognitive deficits (Nebes et al., 2009), and lower levels of life satisfaction (e.g., Paunio et al., 2009: Ness and Saksvik-Lehouillier, 2018). Poor sleep quality is moreover characteristic of insomnia, i.e., a sleep disorder that makes it hard to fall asleep, stay asleep, and/or wake up too early, which is common in psychiatric disorders (Dolsen et al., 2014).

Poor sleep quality may reflect a variety of situational factors. Yet, between-person differences in sleep quality tend to be rather stable (e.g., Knutson et al., 2006). Apart from structural conditions that tend to be stable (e.g., work and family factors), dispositional factors, such as personality factors (e.g., Duggan et al., 2014; Gurtman et al., 2014; Stephan et al., 2018) may influence stress susceptibility and lifestyle factors that affect sleep (Stephan et al., 2018). In the present study, we examined sleep quality in relation to time perspective (TP), a trait-like construct that has attracted much interest in recent years and predicted symptoms of distress beyond more traditional personality factors (e.g., Åström et al., 2018a).

## Time Perspective

TP denotes an individual's habitual way of relating to the personal past, present, and future, including thoughts and feelings associated with each temporal frame (cf. Lewin, 1951; Zimbardo and Boyd, 1999). The Zimbardo Time Perspective Inventory (ZTPI; Zimbardo and Boyd, 1999) is a widely used metric to capture individual differences in this regard. The original version of the inventory encompasses five subscales: Past Positive involves a positive, warm, and nostalgic view of the past, Past Negative captures a negative and aversive view of the past, Present Hedonistic reflects a live-for-the-moment-attitude toward the present, i.e., pleasure seeking for the moment, without much concern of future consequences of current behaviors. Present Fatalistic reflects a hopeless and helpless view of the present, where current behaviors are considered as irrelevant to future consequences. Future, finally, captures a broad positive orientation toward the future including optimism and striving for goals and future rewards. A revised version (S-ZTPI; Carelli et al., 2011) additionally includes a separate Future Negative subscale, which reflects a broadly aversive view of the future, characterized by negative expectations and worry of future consequences.

Consistent with the theoretical framework behind ZTPI (Zimbardo and Boyd, 1999), TP has been associated with a wide range of variables including psychiatric disorders (Oyandel and Buela-Casal, 2014), perceived stress (Papastamatelou et al., 2015), obesity, and smoking (e.g., Guthrie et al., 2013), results often demonstrating that biases on some select ZTPI dimension is critical. Support for the distinction between Future (Positive) and Future Negative was provided by findings of differential associations of the two scales with anxiety (Åström et al., 2018a), perceived stress (Rönnlund et al., 2018), maladaptive coping styles (Blomgren et al., 2016), and measures of well-being (Rönnlund et al., 2017). As recently demonstrated by Stolarski et al. (2021), the two aspects of the future TP also showed a differential association with aspects of the BIS/BAS (inhibition/activation) model of temperament, such that Future Positive is mainly driven by BAS-Drive whereas Future Negative is anchored in BIS.

In common with sleep quality, measures of TP are also associated with measures of life satisfaction (e.g., Desmyter and De Raedt, 2012; Sailer et al., 2014). The TP biases related to life satisfaction seem to involve biases, or deviations, across several of the TP dimensions. Hence, in this case, the overall score profile is worth considering additionally to the subscale scores. To capture the totality of TP biases, Stolarski et al. (2011) proposed a measure known as Deviation from a Balanced Time Perspective (DBTP), computed as the total (squared) difference between an individual's score profile and a proposed optimal, or balanced, ZTPI score profile (Zimbardo and Boyd, 2008). This optimal score profile is characterized by low scores on Past Negative and Present Fatalistic, high score on Past Positive, and moderately high scores on Present Hedonistic and Future. Theoretically, proximity to the ideal values may reflect a better ability to switch between temporal dimensions in an adaptive way (Zimbardo and Boyd, 2008). Importantly, the DBTP measure showed substantial relationships with several major psychological factors, apart from measures of well-being (for a review, see Stolarski et al., 2020), including level of perceived stress (Rönnlund et al., 2018), trait mindfulness (Stolarski et al., 2016), and general cognitive ability (Zajenkowski et al., 2016; Rönnlund et al., 2018). Thus, biases in specific TP dimensions, as well as deviations across all TP dimensions, should be considered when associations with other variables are investigated.

### Time Perspective and Sleep Quality

Several theoretical models acknowledge a major role of thought processes in sleep disorders. In a widely cited model of insomnia by Harvey (2002), for example, excessive negatively toned cognitive activity, including rumination and worry, is considered as a central factor (e.g., Carney et al., 2010; for a review of the empirical evidence, see Hiller et al., 2015). Rumination and worry differ in terms of content; similarly to Past Negative, rumination concerns negatively oriented thinking about the past (Nolen-Hoeksema et al., 2008), while worry is a form of negative thinking toward the future (cf. Future Negative; Watkins et al., 2005; Nolen-Hoeksema et al., 2008). In support of a direct relation between the foregoing TP dimensions, rumination and worry, Åström et al. (2018a) found that Past Negative was the main predictor of rumination, whereas Future Negative was the main predictor of worry. Previous researchers argued that rumination and worry, often directed toward sleep experience itself, are key perpetuating factors in insomnia (Hiller et al., 2015). However, Rönnlund and Carelli (2018b) reasoned that temporal biases in the broader (TP) sense, a predominant Past Negative Future Negative orientation in particular, might be associated with sleeping problems, even in non-clinical samples.

To examine TP and sleep-related problems, Rönnlund and Carelli (2018b) examined data for a relatively large sample of older adults (*N* > 400). The participants responded to a questionnaire about sleep quality and daytime sleepiness (Karolinska Sleep Questionnaire; Åkerstedt et al., 2008), S-ZTPI, and rated happiness and life-satisfaction. In line with the predictions, Past Negative and Future Negative were the TP dimensions most strongly predictive of poor sleep quality. Present Fatalistic additionally showed a small significant positive association with sleep quality in bivariate analyses but not in analyses including all six ZTPI subscales. None of the other scales were related to sleep quality. A highly similar pattern was evident for the measure of daytime sleepiness, i.e., Past Negative and Future Negative were the unique predictors. Finally, path analyses were consistent with a mediational model whereby poor sleep quality accounted for part of the association between DBTP and ratings of well-being.

Whereas the study by Rönnlund and Carelli indicated that poor sleep is mainly related to specific TP biases, this is at odds with a prior study by Vranesh et al. (1999) that reported moderate to strong (*r* = 0.54 – 0.61), positive associations between all five of the original ZTPI scales and the measures of sleep quality. Vranesh et al. took this pattern to suggest a more global link between TP and sleep problems, concluding that: “concerns about time, regardless of the specific nature of individuals' time perspective, and certain sleep problems are significantly related” (Vranesh et al., 1999, p. 23). However, some of these results are surprising in the light of other findings in the TP literature. In particular, the positive association between Past Positive and sleep problems is counterintuitive. In other studies, Past Positive was, for instance, associated with lower levels of stress (Papastamatelou et al., 2015; Rönnlund et al., 2018) lower levels of depressive symptoms (Åström et al., 2018a), and more favorable ratings of well-being (Rönnlund et al., 2017), i.e., factors usually associated with better, rather than worse, sleep quality.

Rönnlund and Carelli (2018b) speculated that a difference in sample composition (Vranesh et al. included a sample of 137 younger students rather than old adults) might account for part of the discrepant outcomes between their study and that by Vranesh et al. (1999), suggesting that future studies should examine this possibility. Additionally, the authors pointed to the need of controlling another sleep-related factor, namely chronotype. Chronotype, or circadian preference, refers to inter-individual differences in preference of timing of the sleep-wake cycle, ranging from early (morningness) to late (eveningness). The need to establish an association of facets of TP and sleep quality beyond chronotype is motivated by findings of a significant relation between chronotype and sleep quality on the one hand and a significant relation between chronotype and TP on the other. More specifically, morning preference has been associated with better sleep quality (e.g., Rique et al., 2014; Hu et al., 2016) and a future-focused TP (e.g., Milfont and Schwarzenthal, 2014), whereas eveningness was related to a more present-focused TP (Stolarski et al., 2012; Milfont and Schwarzenthal, 2014). Hence, chronotype represents a threat to the internal validity in studies of TP and sleep quality that should be adjusted for.

A third study by Borisenkov et al. (2019) accommodated some of the suggestions by Rönnlund and Carelli (2018b). A younger sample (age range 15–25 years) was included. The potential influence of chronotype was additionally adjusted for, in the sense that a measure of chronotype was entered together with sleep quality in separate regression analyses, i.e., with each ZTPI scale as the regressor and other variables as the predictors. These analyses revealed that poorer sleep quality (higher scores on the Pittsburgh Sleep Quality Index), was a predictor of higher scores on Past Negative and Present Fatalistic, as well as a less balanced TP (higher DBTP value), and was therefore in line with the results by Rönnlund and Carelli (2018b). Contrary to the results by Vranesh et al. (1999), but in common with Rönnlund and Carelli, Past Positive and Present Hedonistic were not predictive of poorer sleep quality, although Future Positive was associated with *better* sleep quality.

In spite of the improvement on aspects of prior studies and a large sample (> 1,000 participants), the study by Borisenkov et al. has some limitations. Each of the ZTPI subscales was examined separately, but some of them show moderate inter-correlations. For example, the negatively valenced subscales correlate and may to some extent reflect negativity regardless of temporal direction. This influence may be controlled in multivariate analyses (e.g. regression analyses) to determine a potential unique association with individual scales and sleep quality Additionally, the results, as presented, cannot be used to determine the extent to which TP and chronotype contribute independently to sleep quality. Neither were zero-order correlations of measures of TP, chronotype and sleep quality presented, which would have been informative in this regard. Finally, Borisenkov et al. used the original version of the ZTPI, lacking the Future Negative dimension, which, as noted, was the most prominent predictor of sleep quality in the study by Rönnlund and Carelli (2018b).

Motivated by the discrepant findings in the literature, and differences between studies concerning measures, analytic strategies and sample characteristics, we set out to further examine the relationship between TP and sleep. A young-adult sample similar to that in Vranesh et al. (1999) was included. We used the version of the ZTPI (S-ZTPI) that differentiates positive and negative future dimensions and unlike the previous study by Rönnlund and Carelli (2018b) we additionally included a measure of chronotype for purpose of control. Based on previous studies, we expected that morningness would be associated with better sleep quality and share some variance with TP in the prediction of sleep quality, and that measures of TP would predict differences in sleep quality beyond chronotype. A second aim was to provide a test of conceptual replication of the path model in Rönnlund and Carelli (2018b), by which the expected relationship between DBPT and life satisfaction is mediated by sleep quality.

## Methods and Materials

### Participants

In total, 386 young adults [*M* = 24.6 years, *SD* = 4.87, range 18–40 years, mainly university students (> 95%) in Umeå, Sweden (*n* = 305)], Ludwigshafen and nearby areas in Germany (*n* = 81) took part in the study. Of the participants, 271 were women, 112 men and one “other,” with two values missing for gender. Participants either filled out the included questionnaires by paper and pencil or through an online-survey. The study protocol was approved by the regional ethics board in Umeå and all of the participants provided informed consent prior to filling in the questionnaires.

### Intruments

#### Swedish Zimbardo Time Perspective Inventory (S-ZTPI)

S-ZTPI contains 64 items. Each item is a statement concerning view of/attitudes to time. The questionnaire differs from the original inventory of 56 items (Zimbardo and Boyd, 1999) in that it differentiates positive and negative aspects of the future TP, by adding eight new items to the inventory for the Future Negative scale (two original items for the unitary Future are in addition assigned to Future Negative, while the remaining items form a Future Positive scale). Confirmatory factor analyses provided support of the proposed six-factor structure and internal consistencies ranged from 0.65 to 0.94 across subscales (Carelli et al., 2011). The participants are requested to rate how characteristic each statement is of his/her own view, on five-point Likert scale from very uncharacteristic (coded as 1) to very characteristic (coded as 5). S-ZTPI items belong to one of six subscales: Past Negative (e.g., “Painful past experiences keep being replayed in my mind”), Past Positive (e.g., “Familiar childhood sights, sounds, smells often bring back a flood of wonderful memories”), Present Fatalistic (e.g., “Fate determines much in my life”), Present Hedonistic (e.g., “I believe that getting together with one's friends to party is one of life's important pleasure”), Future Negative (e.g., ”To think about my future makes me sad”) and Future Positive (e.g., “When I want to achieve something, I set goals and consider specific means for reaching those goals”). The subscale scores are computed as the average of rating (1–5) across items.

For the German participants we used the German 56-items version (Reuschenbach et al., 2013). Moreover, the Future Negative items were added to the German scale after being translated forward and backward.

To capture TP biases across all of the TP dimensions, we computed Deviation from a Balanced TP (DBTP; Stolarski et al., 2011). We used the revised version of the formula in Rönnlund et al. (2017) that takes the Future Positive vs. Negative distinction into account:

$$\begin{array}{l} \sqrt{{\left(\text{oPN}-\text{ePN}\right)}^{2}+{\left(\text{oPP}-\text{ePP}\right)}^{2}+{\left(\text{oPF}-\text{ePF}\right)}^{2}+{\left(\text{oPH}-\text{ePH}\right)}^{2}+{\left(\text{oFP}-\text{eFP}\right)}^{2}+{\left(\text{oFN}-\text{eFN}\right)}^{2}} \end{array}$$

where o = optimal score and e = empirical (i.e., observed) score. In accord with several previous studies (Stolarski et al., 2011; Rönnlund et al., 2017), optimal scores were set to: oPN = 1.95, oPP = 4.6, oPF = 1.5, oPH = 3.9, oF/oFP = 4.0, and oFN = 1.8.

An alternative way to estimate DBTP was suggested recently by Jankowski et al. (2020). The arguments raised by Jankowski and colleagues is that the original formula in Stolarski et al. (2011) should be adjusted to include extreme points for three dimensions oPN (=1), oPP (=5), oF (= 5) and an optimal value for PH = 3.4. To examine the possibility that the revised formula would give a different result to those first reported, we recomputed DBTP (the revised formula did not include Future Negative, but, by analogy, oFN was set to (1) and re-run subsequent analyses involving DBTP). The results were highly similar to those presented (i.e., the same patterns of significant direct/indirect effects). We therefore report values based on the original formula for S-ZTPI in Rönnlund et al. (2017).

#### Pittsburgh Sleep Quality Index (PSQI)

PSQI (Buysse et al., 1989) is a widely used questionnaire to assess sleep quality. PSQI consist of 19 items concerned with different aspects of sleep disturbances over the past month. The items generate seven “component” scores: subjective sleep quality, sleep latency, sleep duration, habitual sleep efficiency, sleep disturbances, use of sleeping medication, and daytime dysfunction. The sum of scores for the aforementioned components are added to a global score/index, considered as a global measure of sleep quality, a higher score indicating poorer sleep quality. Adequate values of internal homogeneity, consistency, and test-retest reliability were observed in the original study (Buysse et al., 1989). Here we used Swedish [Forsknings och utvecklingsenheten (FoU), 2018] and German (Hinz et al., 2017) translations of the instrument.

#### Satisfaction With Life Scale (SWLS)

SWLS is a five-item questionnaire developed by Diener et al. (1985) to assess the degree to which the individual's life is perceived as satisfactory. Each of the items (e.g., “In most ways my life is close to my ideal”) is rated on a seven-point Likert scale (1 = strongly disagree, 7 = strongly agree). SWLS has shown adequate construct validity including positive correlations with measures of positive affect and alternative measures of subjective well-being, as well as negative correlations with measures of psychological distress (Pavot and Diener, 1993). High internal consistency was further reported for the Swedish (i.e., α = 0.88; Hultell and Gustavsson, 2008) as well as the German (i.e., α = 0.92; Glaesmer et al., 2011) translations of the instrument.

#### Reduced Morningness-Eveningness Questionnaire (rMEQ)

We used Swedish (Danielsson et al., 2019) and German (Randler, 2013) translations of the reduced Morningness-Eveningness Questionnaire (Horne and Östberg, 1976; rMEQ Adan and Almirall, 1991) to assess chronotype. The questionnaire contains five items assessing individual differences in morningness-eveningness preference (“What time would you get up if you were entirely free to plan your day?”). The German version of rMEQ was strongly associated with an alternative measure of chronotype (Composite Scale of Morningness-eveningness) and has shown acceptable internal consistency (α = 0.72; Randler, 2013; α = 0.68 in Danielsson et al., 2019).

### Statistical Methods

Pearson's *r* was used to evaluate bivariate associations. Linear regression analyses were employed to test models involving scores on the six S-ZTPI subscales as predictors of PSQI score. A mediation analysis was performed to test a hypothetical model whereby DBTP lowers life satisfaction through impaired sleep quality. A bootstrap procedure involving 3,000 bootstrap samples was used to establish 90% confidence bias-corrected confidence intervals (BCIs) for estimates. IBM SPSS 26 and AMOS were used for the statistical analyses.

## Results

### Time Perspective and Sleep Quality

Descriptive data for study measures and zero-correlations are presented in Table 1. The mean score for the PSQI was 7.12 and 63% of participants in the sample scored above the cutoff (PSQI = 5) used to distinguish poor from good sleepers in the original study. The sample means for S-ZTPI subscales were similar to those in the sample used for development of the inventory (Carelli et al., 2011). The mean for the measure of life satisfaction was comparable with that in other studies involving healthy samples.

**Table 1:** Descriptive statistics and zero-order correlations of the study variables.

	***M***	***SD***	**1**	**2**	**3**	**4**	**5**	**6**	**7**	**8**	**9**
1. Sleep quality^a^	7.12	3.61	–								
2. Past Negative	2.89	0.74	0.34^***^	–							
3. Past Positive	3.62	0.69	−0.23^***^	−0.35^***^	–						
4. Present Fatalistic	2.48	0.55	0.27^***^	0.35^***^	−0.05	–					
5. Present Hedonistic	3.22	0.54	0.14^*^	0.08	0.08	0.34^***^	–				
6. Future Negative	2.98	0.61	0.32^***^	0.57^***^	−0.20^**^	0.27^***^	−0.09	–			
7. Future Positive	3.44	0.58	−0.19^**^	−0.12^*^	0.11^*^	−0.37^***^	−0.28^***^	0.09	–		
8. DBTP	2.70	0.75	0.38^***^	0.66^***^	−0.61^***^	−0.13^**^	0.39^***^	0.62^***^	−0.10	–	
9. Chronotype	14.73	3.23	−0.21^**^	−0.13^*^	0.10	−0.08	−0.13^*^	−0.05	0.30^***^	−0.13^*^	–

a *A higher PSQI score reflects poorer sleep quality, DBTP, Deviation from a balanced time perspective*,

* *p < 0.05*,

** *p < 0.01*,

*** *p < 0.001*.

All six ZTPI subscales were significantly associated with PSQI, but in different directions (i.e., positive for Past Negative, Present Fatalistic, Present Hedonistic, and Future Negative, but negative for Past Positive and Future Positive). Moreover, chronotype (higher rMEQ scores indicating morningness) was significantly associated with a couple of S-ZTPI subscales, in particular Future Positive (*r* = 0.30, *p* < 0.001). Scores on rMEQ additionally exhibited a negative correlation with PSQI, indicating that morningness was associated with better sleep quality. Finally, DBTP showed a higher correlation with PSQI than any of the individual S-ZTPI scales (*r* = 0.38, *p* < 0.001) suggesting that biases across several subscales were related to PSQI scores. Preliminary analyses involving two demographic variables (age, sex), indicated that these variables were minimally and non- significantly associated with the outcome measures considered in the present study (i.e., PSQI and SWLS; all correlations ≤ 0.06, *p*-values > 0.20). Hence, they were omitted in further analyses.

To identify potentially unique associations of sleep quality and the S-ZTPI subscales, multiple regression analyses were performed next. Two different models were tested. The first (Model 1) involved the six ZTPI subscales as the predictors of PSQI. In the second model (Model 2) chronotype (rMEQ) was entered in a first step, i.e., prior to the TP dimensions (step 2), to control for this variable. A summary of the results is provided in Table 2.

**Table 2:** Regression analyses of sleep quality^a^.

		**Model 1**			**Model 2**		
		**β**	***t*–value**	**ΔR^**2**^**	**β**	***t–*value**	**ΔR^**2**^**
Step 0/1	Chronotype	–	–		−0.21^***^	−4.22	0.044^***^
Step 1/2	Past negative	0.12^*^	2.04		0.12^*^	1.94	
	Past positive	−0.13^**^	−2.66		−0.13^**^	−2.59	
	Present fatalistic	0.09	1.53		0.10	1.77	
	Present hedonistic	0.09	1.84		0.08	1.65	
	Future negative	0.22^***^	3.64		0.21^***^	3.51	
	Future positive	−0.12^*^	−2.32	0.199^***^	−0.08	−1.49	0.170^***^

a *A higher PSQI score indicates poorer sleep quality*,

* *p < 0.05*,

** *p < 0.01*,

*** *p < 0.001*.

In model 1, the TP dimensions together accounted for nearly 20% of the variance in sleep quality. Past Negative and Future Negative were significant predictors of poorer sleep quality, whereas Past Positive and Future Positive associated with better sleep quality. Results in Model 2 showed that chronotype was a significant predictor in the first step, accounting for 4.4% of the variance in sleep quality; the negative coefficient (β= −0.21) indicating that morningness (higher rMEQ score) was related to better sleep quality (i.e. a lower PSQI score). Critically, the TP dimensions accounted for 17% of the variance in sleep quality over and above chronotype in the second step. To test whether chronotype was also a unique predictor of sleep quality, a third model with the same predictors but reversed entry (i.e., TP dimensions first, chronotype in the second step), was in addition tested. The results confirmed a small but significant increment in variance accounted for, ΔR^2^ = 0.015, *F*_change_ (1, 378) = 0.008, β = – 0.13.

The *r*-value for DBTP and sleep quality (*r* = 0.38; see Table 1) indicated that this measure captures a substantial proportion of the variance (R^2^ = 0.144) in sleep quality accounted for by the individual S-ZTPI dimensions. A final regression analysis involving prior entry of chronotype (cf. Model 2 in Table 2) confirmed that DBTP accounted for a substantial proportion of variance in sleep quality also when chronotype was entered in a first step, ΔR^2^ = 0.129, *F*_change_ (1, 383) = 60.01, β = 0.363, *p* < 0.001. At this stage we also examined a potential interaction between DBTP and chronotype (by including a measure transformed to *z*-scores as well as the cross-product of the measures in a subsequent step), but no such effect was observed (*p* > 0.20).

### Mediation Analyses

**Figure 1:**
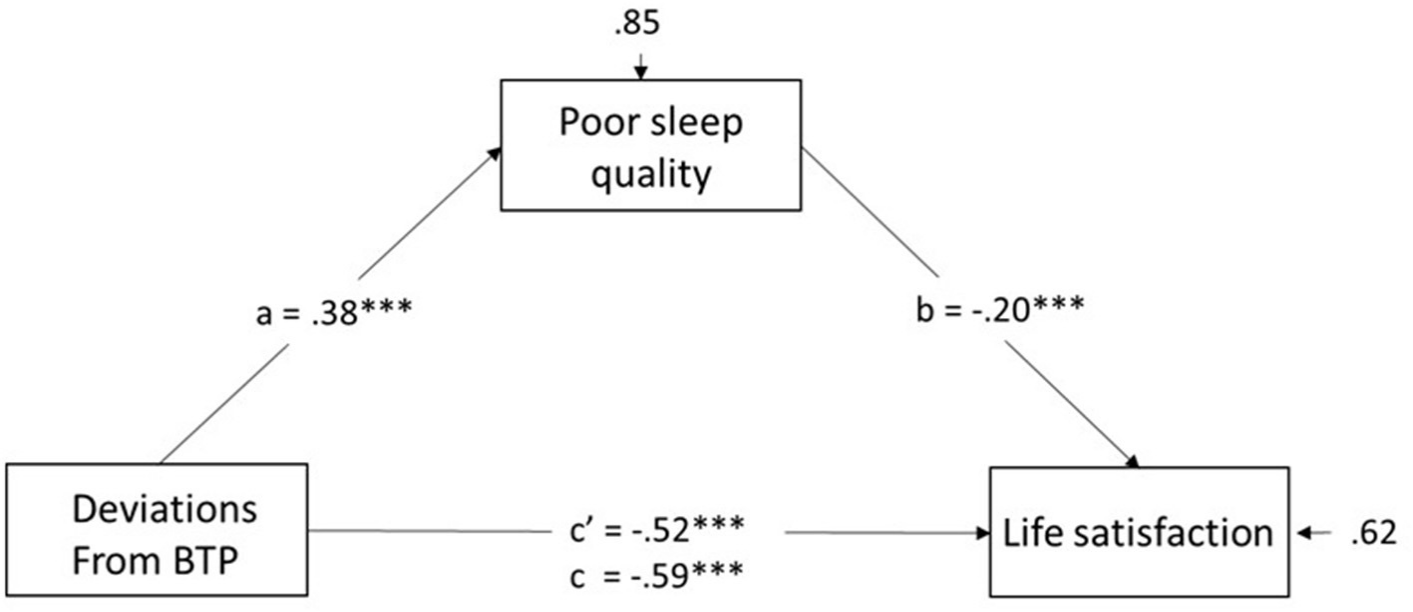
Mediational model where the relation between deviations from a balanced time perspective is mediates by poor sleep quality. c', direct effect of DBTP on life satisfaction. c, total effect of DBTP on life satisfaction. ***p < 0.001.

Having established a significant association between aspects of TP and sleep, with evidence that DBTP captures these associations, we examined a mediational model involving sleep quality as a mediator of the predicted negative relationship between DBTP and the measure of life satisfaction. The model including estimates (β-values) obtained using path analyses is depicted in Figure 1.

DBTP was strongly predictive of SWLS (total effect: c = −0.59). Second, significant estimates in the predicted directions, for the link from DBTP to sleeping problems (0.38) and from sleeping problems to Life Satisfaction (0.20, *p* < 0.001) were observed, results of the bootstrap analyses yielding that the indirect effect (0.075) was significant (*p* < 0.001). Finally, the upper limit of BCI for c', i.e., the direct effect of DBTP on Life satisfaction (−0.588) was lower than the value for the total effect (– 0.592). Thus, the results fulfilled the criteria for partial mediation.

Given some overlap in between TP facets and chronotype in predicting sleep quality (Table 2), we ran a second model with the same basic configuration, but with paths from chronotype to the three other variables to control for the latter variable. In addition, entry of chronotype allowed for the possibility that chronotype itself is a predictor of life satisfaction i.e., beyond TP and sleep quality. The analyses revealed marginally lowered estimates than those in Figure 1, but each remained significant: (a) DBTP to Sleep: β = 0.36, *p* < 0.001; (b) sleep quality to life satisfaction: β = – 0.18, *p* < 0.001; (c) DBTP to life satisfaction: β −0.51, *p* < 0.002 and the indirect effect of DBTP via sleep quality remained significantly different from zero (*p* < 0.001). Finally, the finding of a significant path from rMEQ to SWLS (β = 0.11, *p* < 0.01) was consistent with a small but significant unique association of rMEQ with life satisfaction (morningness being associated with greater satisfaction with life). Together, the predictors accounted for 39% of the variance in life satisfaction.

## Discussion

The current study aimed to examine the relation between TP and sleep quality in young adults. Overall, the results were largely consistent with the results of the prior study involving older adults (Rönnlund and Carelli, 2018b) and those in Borisenkov et al. (2019). First, we replicated the finding of a significant association between Past Negative and poorer sleep, observed in previous studies (Vranesh et al., 1999; Rönnlund and Carelli, 2018b; Borisenkov et al., 2019). Second, Present Fatalistic showed a small but significant positive association with poorer sleep quality in bivariate analyses (see also Vranesh et al., 1999), but not in multivariate analyses. Finally, in line with Rönnlund and Carelli (2018b), Future Negative was the strongest predictor of poorer sleep quality in the multivariate analyses, once more underlining the need to consider this TP dimension in studies of sleep. Finally, the current study confirmed the utility of using an aggregate TP score, such as DBTP, in order to capture the TP-sleep quality association.

A minor difference from the study by Rönnlund and Carelli (2018b) concerned Past Positive, that was significantly associated with better sleep quality in the present study but not in the former study. Similarly, the bivariate analyses and regression analyses (Model 1) indicated that Future Positive was associated with better sleep quality (cf. also Borisenkov et al., 2019). Thus, if anything, the current findings suggest that more aspects of TP are relevant to sleep quality in young adults. It could be, for example, that Future (positive) individuals, who tend to be morning types, have lower social jetlag (misalignment of biological predispositions in regard to timing of sleep and social requirements; Wittmann et al., 2006) by better adaptations to demands imposed by studies or work, which should promote sleep length and quality. Such restrictions may not apply to the same extent in older (retired) individuals, hence rendering differences in this facet of TP less relevant to sleep behaviors. The total amount of variance in sleep accounted for in the current study (about 20%) was also slightly larger than in the prior study by Rönnlund and Carelli (16%). The possibility that the present measure of sleep quality (PSQI) was simply more sensitive than the measure used in the older sample in Rönnlund and Carelli is an alternative. An age group comparison based on the same set of measures might be required to determine whether age is a moderator of some of the more specific TP-sleep associations. In any case, results by Borisenkov et al. (2019), Rönnlund and Carelli (2018b), and those presented here offers no support of the pattern that all ZTPI dimensions, including those with positive valence, are predictive of *worse* sleep quality (Vranesh et al., 1999).

With regard to chronotype, which, as noted, was not considered in studies other than Borisenkov et al. (2019), the results show a significant association between aspects of TP and chronotype, mainly Future Positive; higher scores were related to morning preference (cf. also Stolarski et al., 2012). In line with previous studies (e.g., Rique et al., 2014), the results additionally suggested that morningness is associated with better sleep quality. Nonetheless, most of the variance in sleep quality accounted for by the facets of TP, or DBTP, remained following adjustment for chronotype, enforcing the conclusion of substantive TP-sleep association beyond chronotype.

A second aim was to investigate the associations between TP, sleep quality and life satisfaction, drawing on the model in Rönnlund and Carelli (2018b). In spite of a difference in sample composition relative to that study (i.e., younger versus older adults) and use of different instruments to assess sleep and well-being across studies, the present results were highly similar to those obtained in the previous study. More specifically, DBTP was a strong predictor of (lower) life satisfaction (see also Stolarski et al., 2020) and the data were consistent with an indirect influence of DBTP on life satisfaction via impaired sleep quality. The analyses were hence consistent with partial mediation of the relation between DBTP and life satisfaction. The relatively small ratio of indirect to total effect (0.075/ 0.592 = 0.126) indicates that many other factors need to be considered to account for the link between DBTP and life satisfaction. Once more, chronotype was identified as an independent predictor, with morningness being related to higher levels of life satisfaction, which is consistent with prior findings of higher levels of positive affect for “morning types” compared with “evening types” (e.g., Biss and Hasher, 2012).

## Limitations and Directions for Future Research

A major limitation of the current study is the cross-sectional design, which presents a poor basis for examining directionality of influence among variables. To substantiate the proposed causal links from TP to sleep quality and life satisfaction, longitudinal assessments of the variables should be undertaken and cross-lagged associations between these variables examined. The prediction would be that changes in TP are more predictive of future changes in sleep quality than vice versa. As noted by Rönnlund and Carelli (2018b), a reversed influence is fully reasonable though, i.e. such that enduring sleep-related problems eventually impair health and increase TP biases.

Another limitation concerns the fact that we used a student sample with minimal variations in major demographic characteristics (e.g., age, educational background) with lack of control of many factors that should ideally be considered in studies of sleep quality (e.g., substance use, physical activity) in addition to objective sleep indicators. Moreover, personality factors, such as Neuroticism, showing overlap with some aspects of TP, for example Past Negative and Future Negative (e.g., Stolarski and Matthews, 2016) and associations with sleep disturbances (e.g., Duggan et al., 2014) were not considered. Testing the extent to which the associations between TP, sleep and life satisfaction observed in the present study hold following control for such factors is an essential goal for future replications of our study as well as considering potential mediators of the TP-sleep associations (e.g., anxiety, worry, acute stress).

Apart from studies involving repeated measurements of the relevant constructs, and more rigorous control for relevant background variables, intervention studies should be important to evaluate the proposed causal links; interventions that prove effective in reducing TP biases might also be expected to alleviate sleep-related problems. In that context, it is interesting to note that mindfulness-based interventions reduced DBTP scores, mainly by reducing Past and Negative TPs (Rönnlund et al., 2019) and improved sleep quality as judged from some other studies (e.g., Black et al., 2015). Effects of the time perspective therapy developed by Sword et al. (2015) would be of interest to examine in relation to sleep, in this regard. Another suggestion for future research is to include measures of rumination and worry together with ZTPI to see if the predicted relationships to facets of TP and sleep quality are observed. Finally, it would be of interest to see to what extent insomnia patients exhibit TP biases in the directions expected on the basis of the current findings.

## Conclusions

The results demonstrated a substantial association between time perspective biases and sleep quality in a young adult sample, and provided a conceptual replication of several main findings in a prior study involving older adults, including significant links between Past Negative, Future Negative TPs and poorer sleep quality. These patterns of findings appear consistent with research emphasizing the role of negatively toned thoughts toward the past and future (e.g., Harvey, 2002). Our findings additionally provided a consistency check concerning the hypothesized role of sleep as a mediator in the relationships between deviations from a balanced TP and life satisfaction. Studies including personality assessments, a longitudinal design as well as studies evaluating interventions to reduce TP biases might be useful to examine the proposed causal links between TP, sleep quality, and life satisfaction further.

## Data Availability Statement

The raw data supporting the conclusions of this article will be made available by the authors, without undue reservation.

## Ethics Statement

The studies involving human participants were reviewed and approved by Regional ethics board, Umeå. The patients/participants provided their written informed consent to participate in this study.

## Author Contributions

All authors were involved in conceptualizing the study. EÅ, WW, LF, AU, and JP collected the data. MR wrote a first draft of the paper and conducted the statistical analyses. All authors were involved in revising the manuscript.

## Conflict of Interest

The authors declare that the research was conducted in the absence of any commercial or financial relationships that could be construed as a potential conflict of interest.
